# Ostrich Plucking in South Africa

**Published:** 1882-12

**Authors:** 


### OSTRICH PLUCKING IN SOUTH AFRICA.

When the feathers are ready to be clipped the birds are either taken singly into a small pen, or a number of them are crowded together so closely in a larger pen that they can do little damage, and the feathers are cut. In the former case the bird is made more docile by taking a small bag, or, what is found more convenient, a toeless stocking, and slipping it over his head. While he is thus blinded and bewildered his plumes are cut, one at a time, about two inches above the root of the quill; these stumps being left to become dry, from two to four months afterward, when the bird is again brought up, they are pulled out with a pair of pincers. Even with these precautions the farmer often comes out of the operation with bleeding hands and torn clothes. Formerly—and it is even yet occasionally done—it was the custom to pluck the plume by hand or with the tweezers, leaving the poor bird bleeding and in pain, vinegar and oil being afterward rubbed on them to alleviate this pain. But a strange retribution followed this cruel process; for, before long, the new feathers grew out distorted and often twisted like a corkscrew, as if every twinge of pain had stamped deformity on the future feather. Out of the cock's wing come a row of twenty-four pure white plumes, sometimes two, three, or even four rows. The first row is known in the trade as "primaries," and from this row forward the color changes by shades into gray and finally to black. The female feathers are not so large or so pure as the male's. The feathers are put away, and sprinkled with pepper or camphor, to keep off insects or moths; but this care usually devolves on the agent who may arrange the feathers for market.

Many persons have been set upon by birds when there was no shelter, not even a tree to run to. In such a case, if the pursued were acquainted with struthious tactics, he would lie down flat on the ground, where the bird finds it impossible to strike him. But even this is no light matter, for some birds in their rage at being baffled of their kick will roll over their prostrate enemy, bellowing with fury and trampling upon him in the most contemptuous fashion. One man who thus attempted the lying-down plan found that every time he attempted to rise, the bird would return and stand sentry over him, till at last, after creeping a distance, he got out only by swimming a pond that bounded one side of the camp.

CENTURY.

